# Diversity Competence and Social Inequalities: Overview and Future Directions

**DOI:** 10.3390/ijerph22111749

**Published:** 2025-11-19

**Authors:** Costas S. Constantinou, Lisa Dikomitis, Eirini Kampriani

**Affiliations:** 1 Basic and Clinical Sciences, University of Nicosia Medical School, Nicosia 1700, Cyprus; 2 Warwick Medical School, University of Warwick, Coventry CV4 7AL, UK

## 1. Introduction

Social inequalities have been subject to research and policy design for a few decades now, with limited positive long-term changes. There is evidence suggesting that social inequalities are difficult to tackle locally and globally because they are not a product of one factor but rather shaped and influenced by multiple forces and at multiple levels. Social inequalities are a global phenomenon, affecting sectors spanning from health, education, and employment to housing. Such inequalities persist across both rich and poor nations. Castedo et al. [1] emphasized that life expectancy gaps within and between countries remain stark and, in some cases, are widening. For instance, between 2011 and 2016, OECD countries recorded growing inequalities in life expectancy, despite overall health improvements. The COVID-19 pandemic further magnified these disparities [2]. In England, deprived areas saw sharp declines in life expectancy, while wealthier regions continued to improve [3]. In the U.S., socioeconomic and racial/ethnic inequalities remain powerful predictors of mortality, limiting gains in longevity [4]. Previous policy attempts were relatively unsuccessful because they focused on downstream outcomes rather than addressing structural drivers such as power distribution, inequalities in various aspects of life (the economy, gender, education, residence, etc.), and social and economic experiences over a lifetime [5,6].

Cultural competence was once thought to reduce social inequalities in regard to many aspects, including health and illness. Although it had a positive effect in healthcare (e.g., improving doctor–patient relationships and patient satisfaction [7,8,9]), it seems that its long-term impact on reducing health disparities has yielded mixed results, possibly because of the complexity and multifaceted nature of social inequalities, as outlined above. Interestingly, in the relevant literature, cultural competence is approached as an umbrella term [10], and, on its own, it is no longer considered the desired way to tackle disparities, resulting in the generation of more concepts. For example, intercultural communication, cultural humility, structural competence, and diversity competence, terms designed to capture different dimensions of a diverse world, which were initially encompassed in cultural competence. Diversity competence is a newly defined term [11] for which appropriate measurement tools are still lacking. In general terms, it refers to the competencies necessary for working effectively with various forms of difference, and it has informed the objective of this Topic. On this note, it has better potential to contribute to reducing inequalities in many sectors of life.

Accordingly, our aim was to collect studies, reviews, and concept papers that would move beyond culture and address other forms of diversity, such as ethnic background, religion, socio-economic status, age, gender identity, and disabilities, contributing to the existing scholarship indicating the multidimensional nature of social inequalities.

Before discussing the published papers, let us first outline the characteristics of the Topic and the published papers. The Topic welcomed innovative contributions on social inequalities, inequalities in healthcare, and diversity competence based on research from a range of methodological orientations, considering research articles, reviews, and concept papers. Five journals participated: European Journal of Investigation in Health, Psychology and Education; Healthcare; Societies; Sustainability; and the International Journal of Environmental Research and Public Health.

The Topic generated a great deal of interest, resulting in a rather competitive context for publication. It attracted 97 submissions, 25 of which were accepted for publication, leading to an acceptance rate of 25.77%. The papers discussed a variety of subjects, employing different methodologies and approaches from different countries from around the world. Specifically, the samples varied depending on the type of research, and the methodologies were quantitative, qualitative, and scoping and systematic reviews. The studies focused on subjects in various countries, such as Slovakia, the U.S., Chile, Italy, Spain, Thailand, South Korea, China, Sri Lanka, Australia, New Zealand, Indonesia, Vietnam, and OECD and BRICS-T (Brazil, Russia, India, China, South Africa, and Turkey) countries, showing that this topic has global attraction and implications. We synthesized the findings and insights from these published papers to map the levels of social inequalities and diversity competencies, resulting in the seven themes discussed below.

## 2. A Thematic Discussion of the Published Articles

### 2.1. Multicultural Competence, Leadership, and Education

Several studies highlight the importance of developing multicultural competence as a critical response to diverse societies. Laeheem et al. [12] studied youth leadership in Thailand and emphasized six interlocking competencies for multicultural leadership, ranging from awareness of cultural diversity to flexibility and adaptability to awareness of ethical grounding. These skills were positioned not merely as individual traits but as strategic tools in diverse sociocultural environments. Another study conducted in Thailand—this time by Choompunuch et al. [13], analyzing pre-service teachers’ training in the country—echoed this emphasis, identifying cultural awareness, cultural knowledge, and personal skills as being essential for effective pedagogy. Woo and Cho’s study [14], focusing on anti-hate education in South Korea, complemented these perspectives by situating multicultural competence within critical pedagogy. By teaching students to analyze cultural texts critically, they demonstrated how formal education could cultivate active resistance to hate speech and discriminatory narratives. In a broader context, Constantinou and Nikitara’s [15] systematic review of Delphi studies uncovered the basic competencies that healthcare professionals require in order to work appropriately with patients from different social and cultural backgrounds, indicating the necessity of life-long learning education and training. These competencies were reflection, be educated, showing interest and praising, empathy, and the ability to collaborate for therapy.

Together, these studies underline education’s potential to promote multiculturalism and place emphasis on other aspects of multicultural competence, such as leadership, critical citizenship, and partnership with patients. Furthermore, multicultural competence seems to be context-dependent in the sense that in some settings, it was about inter-ethnic coexistence, while in others, it was about equipping youth to confront structural hate.

### 2.2. Structural Inequalities in Health and Living Conditions

Reviewing the published papers revealed that health disparities featured strongly in studies on marginalized populations, illustrating how exclusion operates through environmental conditions, systemic neglect, and cultural dissonance. Ihnacik et al.’s [16] study on Roma communities in Slovakia revealed that poor settlement location affected access to safe water and sanitation, rendering people vulnerable to infections. Therefore, in this case, the issue did not directly relate to ethnicity and instead pertained more closely to geographical location and integration. The issue of integration was also raised in Pham’s [17] study of rural Vietnam indicating the importance of a shift in mindset to appropriately include people with disabilities in decision-making. Inclusivity was also necessary in the case of Australia and its Aboriginal communities [18], who require better access to healthcare and more effective cultural connection. Māori and Pasifika endometriosis patients in New Zealand [19] face inadequacies in healthcare delivery, underscoring the need for better culturally congruent care. Structural issues in healthcare were also identified in Reid et al.’s [20] study of maternal health data in Florida, where, for example, misclassification of health records threw the quality of care into question and distorted the visibility of inequalities.

### 2.3. Economic Inequality, Housing, Institutional Quality, and Perceived Work Flexibility

The structural inequalities are directly linked with income and wealth. Economic inequality—manifested through housing, institutional structures, and conditions of work—emerged as a recurring concern in several studies. In their study of OECD countries, Ünalan et al. [21] found that rising housing prices reduced inequality in low-income countries with higher homeownership rates but had negligible or adverse effects in wealthier contexts. Similarly, Lee’s [22] study of U.S. immigrants showed that asset ownership—not merely income—enabled access to healthcare, with nuanced differences across ethnic groups. Extending the analysis to institutional dimensions, Uzar’s [23] work on BRICS-T countries demonstrated that property rights, corruption control, and freedom of the press could mitigate inequality, even in the absence of rapid economic growth.

Interestingly, Lay-Raby et al. [24] examined how inequality was also reproduced within the labor market. Their study on Chile revealed that the distribution of perceived work flexibility is far from equal: “flexibility is a stratified experience,” wherein women are more likely to access only partial flexibility and workers with higher educational attainment report lower perceptions of flexibility. Occupation emerged as the strongest predictor, with employers enjoying full autonomy, while dependent workers remained constrained.

These studies reveal that inequality is not monolithic. Its mechanisms operate through housing markets, asset ownership, institutional quality, and labor conditions alike. While some studies highlight the role of economic and institutional safeguards, others reveal how workplace arrangements can mirror broader structural hierarchies. Collectively, they depict a spectrum of pathways through which economic structures perpetuate—or, in some contexts, mitigate—inequality.

### 2.4. Exclusion and Inclusion in Education

Education is a major area where inequalities manifest and resilience is cultivated. For example, Ewuoso and Ogundiran’s [25] study on internal exclusion in African education introduces the concept of being “excluded while included,” operating through mechanisms like othering, epistemic de-rooting, and linguistic hierarchies. A study on educational poverty during the COVID-19 pandemic in Italy [26] highlighted differences in teachers’ attitudes toward inclusion. While some educators acted creatively to support students with special needs, others conformed passively, reflecting varied capacities to counteract deprivation. Interestingly, the educators’ attitudes were different during the post-pandemic period, changing from being atomistic to more collaborative. Fernández-Arias et al. [27] focused on the digital competence of rural teachers in Spain, highlighting how teacher training could bridge rural–urban divides, suggesting that digital skills were crucial for inclusive pedagogy in depopulated regions. Interestingly, Yan and Gai [28] explored academic resilience among low-SES students in Chinese families and identified protective factors that enable disadvantaged students to excel, such as achievement motivation, positive emotions, and supportive relationships.

These studies again reveal the complexity of the issue and that social inequalities are neither monolithic nor set in stone. While African and Italian studies have highlighted structural and attitudinal exclusions, Spanish and Chinese studies have underscored resilience and potential transformation. Together, they suggest that exclusion and resilience are two sides of the same coin, revealing both the barriers and possibilities embedded in educational systems.

### 2.5. Inequalities in Representation, Narrative, and Data

Beyond institutions and access, inequalities are reproduced in how narratives are framed and data are collected. Bragg et al. [29] studied food justice in the U.S., criticizing dominant narratives that individualized hunger while ignoring systemic drivers of food insecurity. The authors suggested reframing narratives as being essential for policy change. Along similar lines, Higashida et al. [30] investigated social-work representations in Sri Lanka and revealed tensions between local and Western professional frameworks, showing how discourse shapes practice. Narratives and discourse are ways of presenting information; in accordance with this notion is Reid et al.’s [20] research on health data use in Florida, which illustrated how inaccurate data obscured disparities, undermining accountability.

The above studies collectively demonstrate that representation—whether in policy narratives, data systems, or professional discourses—constitutes a powerful site of inequality. Unlike structural barriers in health or education, these forms of exclusion are epistemic, shaping what is visible and actionable.

### 2.6. Minority Stress, Altruism, and Social Relations

Another thread concerns how inequalities are experienced at the individual and relational level. Cisek and Rogowska [31] showed how LGBTQA minority stress in Poland was directly linked to depression, particularly for individuals with dual marginalized identities. Despite the stress and its implications—features also highlighted in other studies, such as Rasali et al.’s work [32]—it seems that people do not passively experience inequalities but instead they adjust and develop through social relationships. For example, Liu et al. [33] revealed that low-SES Chinese students were more altruistic, a characteristic mediated by affective empathy rather than cognitive empathy. This counterintuitive finding challenged deficit-oriented views of disadvantaged groups. Yusra and Lestari [34] illustrated how interethnic relations in Indonesia could foster sustainability, suggesting that diversity itself was a resource.

These papers differ in their accounts of social dynamics. While some show how inequality generates psychological stress, others highlight empathy and mobility as pathways for solidarity. Together, they suggest that inequalities do not merely fracture societies; they also provoke new forms of connection and resilience.

### 2.7. Culturally Responsive Public Services for Community Well-Being

A couple of studies highlight the importance of public service responses in ensuring community well-being. For instance, Sun et al.’s [35] township cultural service study highlights how access to facilities, cultural talent, management, and culturally relevant activities determines satisfaction with rural cultural programs in China. Similarly, Ford et al. [36] showed that structural barriers, mistrust, and cultural differences affected acceptance of COVID-19 vaccination among Asian Americans. Furthermore, the study indicated that some Asian Americans might be difficult to approach and include due to language barriers and cultural norms. In response, the authors suggest creating resources and programs to address misconceptions. Interestingly, Higashida et al. [30] maintained that work with minorities underscores persistent inequalities. Specifically, during the COVID-19 pandemic, social workers faced heightened demands in relation to addressing unemployment, food insecurity, domestic violence, and isolation—often through using community-based responses to sustain vulnerable groups.

These findings suggest that public services—whether cultural or health-related—must be tailored to specific communities, considering their unique challenges, traditions, and perspectives, in order to promote equity, accessibility, and meaningful engagement.

## 3. Future Directions in Research and Policy

Our thematic discussion of the published papers in this Topic shows the complexity of social inequalities at the global level and that competence in diversity should be approached as a multi-dimensional set of skills and knowledge at individual, organizational, and social and policy levels. This means that the issue does not have one factor or cause and that competence is not one set of knowledge and skills in one context. Therefore, reducing inequalities requires a deep understanding of the causes, targeted policy actions, mastered diversity competencies at multiple levels, and having the skill to adapt to and understand changing contexts.

Future research must move toward integrative frameworks that bridge structural, cultural, and relational dimensions of inequality. First, we require comparative longitudinal studies to examine how multicultural competences evolve and impact institutions over time. Second, health research should embed culturally safe practices into systemic reforms, not just localized interventions. Third, economic inequality work should further interrogate the interplay between assets, institutions, and cultural capital. Fourth, education studies would benefit from linking resilience-building to structural transformation, avoiding the trap of individualizing responsibility. Finally, intersectional analyses combining quantitative and qualitative methods are essential to reveal how inequalities intersect across axes of race, class, gender, sexuality, and geography.

Advancing diversity competence and equity requires not only situational sensitivity but also conceptual and operational advancements in the definition and measurement of ‘diversity competence’ at the individual, organizational, and societal levels. Accordingly, professionals could be trained in diversity competencies, while organizations should have well-embedded cultures and structures of valuing diversity and tackling any incidents of biases and stereotypes. Societies may establish embracing educational systems and bureaucratic or structural pathways of inclusivity in all sectors of life.
